# The non-fatal disease burden caused by type 2 diabetes in South Africa, 2009

**DOI:** 10.3402/gha.v6i0.19244

**Published:** 2013-01-24

**Authors:** Melanie Y. Bertram, Aneil V.S. Jaswal, Victoria Pillay Van Wyk, Naomi S. Levitt, Karen J. Hofman

**Affiliations:** 1 MRC/Wits Rural Public Health and Health Transitions Research Unit (Agincourt), School of Public Health, Faculty of Health Sciences, University of Witwatersrand, Johannesburg, South Africa; 2 Department of Public Health, University of Oxford, Oxford, UK; 3 Burden of Disease Research Unit, Medical Research Council of South Africa, Tygerberg, Western Cape, South Africa; 4 Department of Medicine Health Sciences Faculty, Division of Endocrinology and Diabetes, University of Cape Town; 5 Chronic Diseases Initiative in Africa, Cape Town, South Africa

**Keywords:** burden of disease, diabetes, South Africa, developing country, epidemiology

## Abstract

**Background:**

Increasing urbanisation and rising unhealthy lifestyle risk factors are contributing to a growing diabetes epidemic in South Africa. In 2000, a study estimated diabetes prevalence to be 5.5% in those aged over 30. Accurate, up-to-date information on the epidemiology and burden of disease due to diabetes and its sequelae is essential in the planning of health services for diabetes management.

**Objective:**

To calculate the non-fatal burden of disease in Years Lost due to Disability (YLD) due to diabetes and selected sequelae in South Africa in 2009. YLD measures the equivalent loss of life due to ill-health.

**Methods:**

A series of systematic literature reviews identified data on the epidemiology of diabetes and its sequelae in South Africa. The data identified were then applied to Global Burden of Disease (GBD) methodology to calculate the burden attributable to diabetes.

**Results:**

Prevalence of type 2 diabetes in South Africa in 2009 is estimated at 9.0% in people aged 30 and older, representing approximately 2 million cases of diabetes. We modelled 8,000 new cases of blindness and 2,000 new amputations annually caused by diabetes. There are 78,900 YLD attributed to diabetes, with 64% coming from diabetes alone, 24% from retinopathy, 6% from amputations, 9% from attributable stroke disability, and 7% from attributable ischemic heart disease disability.

**Conclusion:**

We estimate that the prevalence of diabetes is increasing in South Africa. Significant disability associated with diabetes is demonstrated. Some of the attributed burden can be prevented through early detection and treatment.

South Africa is a country which is undergoing rapid epidemiological transition (1). Burden of disease data from 2000 indicate that chronic conditions were already responsible for 30% of the total disease burden (2). Ongoing rapid urbanisation is leading to escalating lifestyle risk factors, such as unhealthy diet and lack of physical activity, which in turn contribute to increasing chronic disease rates (3, 4). Understanding the main contributors to disease burden is essential in the planning of health care facilities and programmes aimed at addressing this growing problem (2).

Diabetes is a growing cause of premature mortality and morbidity worldwide. In 1998, the World Health Organization (WHO) estimated that there were 135 million people with diabetes (5). By 2008, the estimated global prevalence had more than doubled to 347 million (6). This figure is expected to continue to increase. Despite previously being considered a disease of high-income countries, middle-income countries undergoing rapid epidemiological transition are now considered the epicentre of the future diabetes epidemic (7), largely due to an ageing population and the lifestyle changes associated with urbanisation (8). The age pattern of diabetes prevalence is expected to differ between Africa and higher income regions. The majority of diabetes in Africa is prevalent in working-age people, aged between 40 and 60 years, rather than those older than 60 years (9).

Globally, diabetes was estimated to cause 3.96 million excess deaths and 6.8% of all deaths in 2010 (10). Complications associated with diabetes, such as retinopathy and blindness, kidney disease, neuropathy, vascular diseases, and amputation, lead to significant morbidity (7). In 2004, the WHO estimated that diabetes accounted for 19.7 million disability-adjusted life years (DALYs) (11). One DALY represents the loss of the equivalent of 1 year of full health.

Burden of disease data is an important input in evidence-informed policy making (12). In South Africa, this is especially important in the presence of a ‘quadruple burden of disease’, including HIV&AIDS, maternal and child health, chronic disease, and injuries. Though specific costing data are not available for South Africa, across the six richest African countries, the direct and indirect cost of diabetes is estimated to be as much as INT$11431.6 per diabetes case per year (13).

In South Africa, the only previous study on the overall burden of disease attributable to diabetes estimated the burden for 2000 (14). The estimated prevalence of diabetes was 5.5% for South Africans aged over 30, accounting for 4.3% of deaths and a total of 258,000 DALYs.

The aforementioned national burden of disease study for South Africa (SA NBD) was generally unable to prospectively evaluate the non-fatal burden. For a few diseases local data could be used, but for most diseases a ratio was taken between the fatal and the non-fatal component of the DALY for the WHO Africa estimates, and this same ratio applied to mortality in South Africa (15). The current analysis uses a prospective methodology, in line with global estimates, which will increase comparability and increase the data available from the results. Because this methodology is far more data intensive, it presents difficulties in a middle-income setting such as South Africa, where such data are not always available. This study models incidence, prevalence and mortality due to diabetes as well as its sequelae, and provides a summary of diabetes epidemiological data available in South Africa.

## Methods

The methods were largely based on the established Global Burden of Disease (GBD) methodology (16) and use the Years Lost due to Disability (YLD) as the outcome measure (16). This allows for comparability with the previous SA NBD data and GBD data for the sub-Saharan African region. We calculate the YLD attributable to type 2 diabetes and a limited set of sequelae, including blindness due to retinopathy, leg and foot amputations, and the attributable burden to diabetes from cardiovascular diseases.

## Data collection

### Diabetes cases

A systematic review of the literature was performed to identify published prevalence and mortality studies for diabetes in South Africa, from 1990 to present. The search was undertaken in PubMed in June/July 2011. Experts were also contacted for any unpublished work (full details, Appendix 1). Ideally, only data from 2005 onwards would be included in the analysis; however, because we could not be certain that sufficient recent data would be available, we expanded the search dates.

Prevalence studies were included if they were community-based studies, and diagnosis of diabetes was based on the revised WHO criteria for diagnosis of diabetes for a 75 g Oral Glucose Tolerance Test (OGTT), that is, fasting plasma glucose (FPG) > = 7.0 mmol/l and/or 2-h plasma glucose > = 11.1 mmol/l (17). Mortality studies were included if they reported community-based deaths in patients with diabetes. Studies which reported Statistics South Africa vital registration data were not included because we had access to the source data.

### Diabetic sequelae

A systematic review of the literature was performed to identify published prevalence and incidence studies for diabetic retinopathy, amputations, diabetic foot, neuropathy, and kidney failure in South Africa, from 1990 to present. PubMed searches were undertaken in June/July 2011 (full details, Appendix 1). The year 1990 was used as a cut-off for data to ensure we covered as many sequelae as possible. Inclusion criteria were a population- or clinic-based screening study in patients with diabetes. For retinopathy, the time since onset of disease was an inclusion criteria.

### Attributable burden of stroke and ischaemic heart disease

There is well-documented evidence that diabetes mellitus increases the risk of morbidity and mortality due to cardiovascular diseases, namely stroke and ischaemic heart disease (IHD). We attribute some morbidity due to stroke and IHD to diabetes using a population attributable fraction (PAF), which calculates the proportional reduction in disease morbidity or mortality that would occur should risk factor exposure be removed.

The PAF equation is as follows:

$$\text{PAF}=\frac{\text{p}(\text{RR}-1)}{\text{p}(\text{RR}-1)+1}$$

Where p = prevalence of diabetes and RR = relative risk of stroke in people with diabetes.

Currently, in South Africa, no local information is available on the relationship among diabetes, stroke, and IHD. In the Asia Pacific Cohort Studies Collaboration, the relative risk of stroke was 2.04 in diabetic males and 2.0 in females (18). These relative risks are used in the GBD study and have been used in a number of national level burden of disease analyses where no local data exist (19). Applying the PAF to the YLD previously calculated for stroke (20) provides an estimate of the burden of disease attributable to diabetes. IHD burden has not been updated for South Africa for 2009. In place of direct information on disease burden, we applied a ratio to the YLD attributable to stroke, based on WHO estimates of disease burden (21). This enabled an estimation of the attributable YLD from IHD.

## Calculating the burden of disease

### Derivation of incidence and duration using computer-based modelling

The epidemiological parameters required to calculate YLD, incidence, and duration are not always available. DisMod II is a specialised software tool which creates an internally consistent set of epidemiological parameters for a condition given three parameters as inputs (22). In DisMod, remission is defined as ‘cure’, hence no remission is possible when modelling people with diabetes. Accordingly, the data gathered from the literature for prevalence and relative risk of mortality and a remission rate of zero were used as inputs for DisMod, yielding estimates of incidence and duration as outputs.

### Disability weights

Disability weights are a comparative measure of the impact of the complications of illness. No disability weights specific to South Africa were available. For this study, GBD disability weights and, where these were not available, Dutch disability weights were used (23). We did this for consistency with previous GBD estimates and to enable comparisons between the results of the current study and previous studies. For uncomplicated diabetes, a GBD weight of 0.012 for untreated diabetes and 0.033 for treated diabetes were used; for retinopathy, Dutch weight of 0.17 for moderate diabetes and GBD weight of 0.522 for severe diabetes were used (assuming 66% of cases as severe and 33% as moderate, based on data from the Australian burden of disease study, as no data were available for South Africa (24)), and for amputations, GBD weights of 0.102 for toe and 0.300 for foot/leg amputation were used.

## Results

### Literature search results

A total of 117 articles published between 1990 and July 2011 were identified through the search for diabetes prevalence in South Africa. After title and abstract screening, the full text of 16 articles was read. Full details of the literature search results are given in Appendix 2. At this stage, we restricted the years of inclusion to 2005 onwards. Three studies were deemed to contain sufficient information for inclusion (25–27). Raw data were provided by the authors of all studies. One unpublished study, since published, was identified and included in the analysis (28). Overall, we had data from two rural populations, one urban township, and one metro urban population. Data were combined by weighting for the population it represented. The sources of data are shown in Table 1.

**Table 1:** Data used in the epidemiological modelling

Author	Year	No.	Method	Location	Reference
Diabetes prevalence					
Motala et al.	2008	1,025	Cross-sectional survey	Rural KwaZulu Natal, South Africa	(26)
Stewart et al.	2011	1,311	Screening in consecutive primary care patients	Soweto, South Africa	(27)
Groenewald et al.	2009	552	Cross-sectional survey	Rural southern Free State, South Africa	(25)
Levitt et al.	2012		Cross-sectional survey	Cape Town, South Africa	(28)
Retinopathy prevalence					
Rotchford	2002	253	Consecutive patients attending diabetes clinic	Rural KwaZulu Natal, South Africa	(31)
Motala	2001	219	Retrospective analysis of clinical records	Durban, South Africa	(30)
Levitt	1997	300	Random sample of patients attending diabetes clinic	Cape Town, South Africa	(29)
Conradie	1998	311	Retrospective analysis of clinical records	Cape Town, South Africa	(32)
Amputation prevalence					
Levitt	1997	300	Random sample of patients attending diabetes clinic	Cape Town, South Africa	(29)

Twenty-one studies reporting retinopathy were identified, with four giving information on time since diagnosis, which is required to model incidence (29–32). One of these contained two mutually exclusive populations, giving five data points (32). Incidence of proliferative diabetic retinopathy is derived from data on the progression to retinopathy by duration of diabetes (male and female progression is assumed to be the same; insufficient data were available to separate by sex). Using linear regression, an annual incidence of retinopathy was calculated which was then applied to survivors of diabetes by age. The annual incidence calculated was 126 per 100,000 across all ages.

Thirteen articles that discussed diabetic foot disease in South Africa were identified, with only one presenting sufficient information to use in our analysis. Levitt et al. reported 1.4% of patients with diabetes in attending primary care clinics had an amputation of either foot or toe (29). Insufficient information was available to include either diabetic foot or neuropathy. Although information is increasing, data in this area is lacking for South Africa (33).

Of the 15 articles identified through the search for kidney failure, one presented sufficient information on renal failure mortality (34). Our search revealed no information on incidence or prevalence of kidney failure for South Africa, so it was not possible to include it as a sequela in this analysis. Left untreated, renal failure is largely fatal; it contributes minimally to the non-fatal burden due to diabetes.

### Burden of disease results

**Fig. 1:**
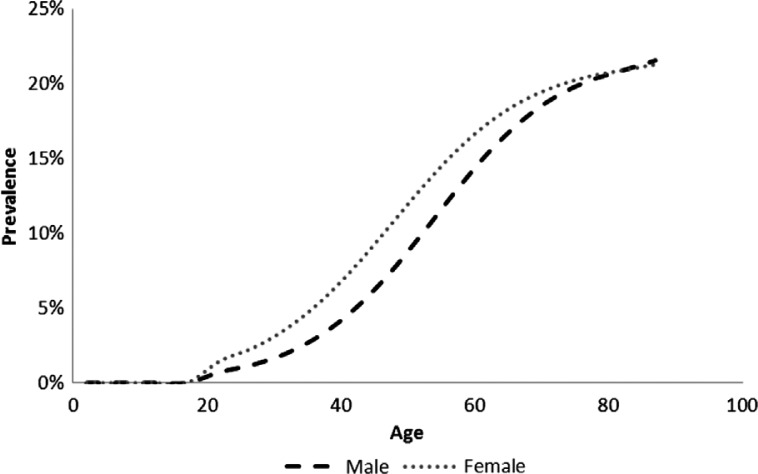
Modelled type 2 diabetes prevalence in South Africa, 2009.

The overall prevalence of type 2 diabetes in those over age 30 was estimated at 9.0% (7.4% in men and 10.4% in women), a significant increase over the 5.5% prevalence reported for 2000 (14). This gives a total of 1.97 million cases of type 2 diabetes in South Africa. A secondary finding from the literature search was that 55% of cases are undiagnosed – for South Africa this means that about 1 million people with type 2 diabetes do not know they have it. Modelled age-specific diabetes prevalence is shown in Fig. 1.

As well as 1.97 million people with type 2 diabetes, the model also shows that 115,000 new cases develop each year, along with 7,800 cases of vision impairment due to retinopathy and 2,100 toe or foot amputations due to diabetes (Table 2).

**Table 2:** Modelled incidence of diabetes and sequelae in South Africa, 2008

Age group	Diabetes		Retinopathy		Amputation (foot or toe)	
		
	Male	Female	Male	Female	Male	Female
25–34	7,106	11,853	18	38	0	0
35–44	12,644	17,594	143	213	166	246
45–54	15,419	16,768	493	551	316	386
55–64	10,373	9,238	956	838	246	214
65–74	4,722	4,518	1,260	985	158	140
75+	2,191	3,009	1,182	1,134	94	114
Subtotal all ages	52,455	62,980	4,050	3,760	980	1,100
Total both sex		115,435		7,810		2,080

**Fig. 2:**
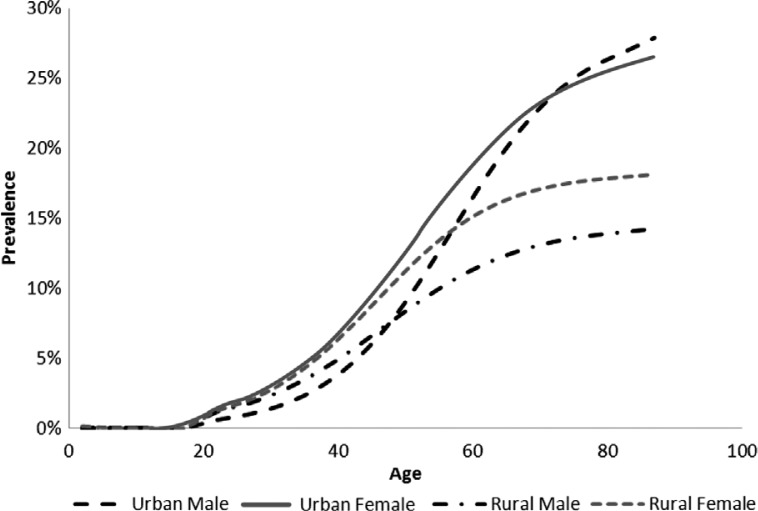
Modelled prevalence of type 2 diabetes in urban and rural South Africa, 2008.

The number of people categorised as urban or rural was last analysed for 2001, when it was estimated that 57.5% of people lived in urban areas and 42.5% lived in rural areas (35). According to our analysis, 35% of people with diabetes live in rural areas and 65% in urban areas, indicating an unequal distribution. In urban areas, among the aged, prevalence is higher in females, whereas in rural areas, prevalence is higher in females than males at all ages (Fig. 2).

A total of 42,919 YLD were estimated as attributable to type 2 diabetes alone, with a further 13,458 arising from retinopathy, 4,527 from amputations, 7,233 from stroke, and 5,577 from IHD, totalling to 73,714 YLD.

A sensitivity analysis was undertaken to look at the impact of the highest and the lowest prevalence measures on the outcomes. By excluding the highest prevalence study (28) overall diabetes prevalence drops to 5.2% in line with a decade previously and results in a halving of the attributable YLD, to 23,000. Excluding the lowest prevalence study for both urban and rural areas (25, 27) had the opposite impact, causing prevalence to increase to 12.1% overall, resulting in 53,000 attributable YLDs.

## Discussion

Our estimates indicate that the burden of disease due to diabetes may have grown significantly since the previous South African burden of disease study in 2000 (14). Prevalence of diabetes in people over age 30 has potentially increased from 5.5 to 9.0% since the previous estimates. This may partly be a reflection of the prevalence data used in 2000, which was largely from the early 1990s, so the increase seen may have occurred over almost two decades. The assumption made in the previous study that diabetes prevalence in rural areas was half that in urban areas was likely too conservative and contributes to the differences between the previous study and the current study results. Due to ethnic variations in diabetes prevalence in South Africa, there may be continuing issues around data representativeness. In particular, there are no recent studies in either Indian or Coloured populations. Comparison between the YLD from the SA NBD study in 2000 (14, 15) to the current study was not considered due to the different methodologies used.

Global estimates of diabetes prevalence and burden of disease, such as the International Diabetes Federation prevalence projections and the WHO's GBD study rely on publicly available data and projection modelling. For our analysis, we had access to primary data from various strata of the South African population. Global modelled estimates have generally assumed that risk factors such as obesity will remain constant (5, 8). Given that obesity is increasing globally, this assumption is too conservative to accurately predict diabetes prevalence (36, 37). In South Africa, the estimated prevalence of overweight and obesity in 1998 was 29% in men and 56% in women, and that number is thought to have increased significantly since then (38).

Sensitivity analyses indicate the uncertainty within the estimation of diabetes prevalence and burden in South Africa. Given the diversity of the South African population, the use of a single estimate from a non-representative population sub-group to model prevalence is unlikely to provide reliable estimates. Our use of multiple data points to represent urban, peri-urban and rural populations provides the most accurate overview of the national situation in South Africa, given the current data availability in the country. However, given strong ethnic variations in diabetes prevalence indicated in prior literature, the collection of more representative data would provide stronger estimates of disease burden.

The major weakness of the analysis relates to data unavailability. For this reason, we did not incorporate all the sequelae the GBD studies generally recommend. Missing in this study are estimates of cataract, glaucoma, diabetic foot, neuropathy, renal failure, and peripheral vascular disease. Although cataract and glaucoma generally contribute only a small amount to the disease burden, other complications such as diabetic foot, neuropathy, and peripheral vascular disease can each be responsible for up to 5% of the attributable burden to diabetes (19). Furthermore, the sequelae data that exist are less recent than information on diabetes prevalence. Given the rapid increase in diabetes prevalence from 2000 to 2009, it is likely that the epidemiology of the sequelae has also changed during this time.

Data on case fatality rate or relative risk of mortality in people with diabetes in South Africa does not exist. Information from the vital registration system regarding diabetes mortality is not accurate as multiple underlying causes of death are reported with diabetes, for example, cardiovascular diseases, hypertensive heart disease, IHD, and stroke (39, 40). This could result in deaths from diabetes being coded to one of the underlying causes.

The methodological developments which are currently underway within the GBD update, due out in 2013, may have implications for our results. First, methodology will be available to fill gaps using data from elsewhere. This would improve our ability to include sequelae, yet still would not use data specific to South Africa. Second, an update to the disability weights used in the GBD study is anticipated. We do not know how new disability weights will compare to those used in this study. These factors need to be considered when interpreting these findings or comparing with previous and future studies.

In an era of growing obesity, rapid urbanisation, and an ageing HIV positive population in South Africa, this work has highlighted the need for more accurate and complete data with respect to diabetes. The first priority is a diabetes registry. This would be a challenging proposition in the current climate of inadequate informatics programmes even in the most organised provincial health departments. Since half of people with diabetes remain undiagnosed and untreated, a national registry would likely capture only those with known diabetes, but at least this would inform the rollout of South Africa's National Health Insurance Scheme. The second imperative is a nationally representative cross-sectional survey to properly establish the prevalence of diabetes.

This work sets the stage for future analysis on the cost-effectiveness of diabetes prevention and treatment campaigns for South Africa. Whilst it is essential to know the main causes of disease burden, and to be able to quantify the epidemiology, information on the costs and avoided disease burden associated with interventions is also crucial. With the burden of disease information now available, more studies can more thoroughly explore these hypotheses. There is a need to systematise data collection around diabetes and primary health care delivery to facilitate these analyses.

## Conflict of interest and funding

The study was funded by the Bill & Melinda Gates Foundation through the Disease Control Priorities Network (DCPN) project grant to the Department of Global Health at the University of Washington. The study was undertaken under the auspices of PRICELESS SA (Priority Cost Effective Lessons for Systems Strengthening – South Africa) based at the University of Witwatersrand School of Public Health. The funding source had no role in study design, data collection, data analysis, data interpretation, or writing of the report.
